# Supplement use by UK-based British Army soldiers in training

**DOI:** 10.1017/S0007114514001597

**Published:** 2014-08-14

**Authors:** Anna Casey, Jason Hughes, Rachel M. Izard, Julie P. Greeves

**Affiliations:** 1 Consultant in Defence Science, Faber House, Ibstone, BuckinghamshireHP14 3XT, UK; 2 Army School of Physical Training, Fox Lines, Aldershot, UK; 3 HQ Army Recruiting and Training Division, Wiltshire, UK

**Keywords:** Supplements, Military, Training, Performance nutrition

## Abstract

The use of supplements is widespread at all levels of civilian sport and a prevalence of 60–90 % is reported among high-performance UK athletes, including juniors. The prevalence of supplement use among UK-based British Army personnel is not known. The aim of the present study was to establish the point prevalence of supplement use in UK-based British Army soldiers under training (SuTs) and associated staff. A cross-sectional anonymous survey was carried out in 3168 British Army SuTs and soldiers, equating to 3·1 % of regular Army strength, based at eleven Phase 1, 2 and 3 UK Army training sites. Overall, 38 % of the respondents reported current use of supplements, but prevalence varied according to the course attended by the respondents. The number of different supplements used was 4·7 (sd 2·9). Supplements most commonly used were protein bars, powders and drinks (66 %), isotonic carbohydrate–electrolyte sports drinks (49 %), creatine (38 %), recovery sports drinks (35 %), multivitamins (31 %) and vitamin C (25 %). A small proportion of respondents reported the use of amphetamines and similar compounds (1·6 %), cocaine (0·8 %), anabolic androgenic steroids (1·1 %), growth hormone (2·0 %), and other anabolic agents, e.g. testosterone (4·2 %). Logistic regression modelling indicated that, for current users, younger age, being female, smoking and undergoing Officer Cadet training were associated with greater supplement use. This is the first study to investigate the prevalence of dietary and training supplement use in UK-based British military personnel. Self-administration of a wide range of supplements is reported by British military personnel in training, which is at least as great as that reported by those on deployment, and has implications for Defence policy and educational needs.

British Army soldiers under training (SuTs) typically undergo arduous military training for extended periods of time during which they can, at various times, experience significant fatigue, sleep deprivation and difficulty in meeting daily energy requirements, while facing continual assessment within their training environments. Average daily energy expenditures can be very high, reaching in excess of 20 MJ/d during some Phase 1^(^ ^1^ ^)^ and Phase 3 training programmes^(^ ^2^ ^)^, and training is sustained over 3 months, such as recruit Phase 1 training, to 11 months, such as the Commissioning Course for Officer Cadets at the Royal Military Academy Sandhurst (RMAS).

During Phase 1 training, recruits undergo generic military tactical and physical training on a daily basis. Phase 2 training is an extension of Phase 1; it is trade specific and typically less physically demanding. The level and volume of physical training vary between trades and are considerably higher, for example, during Phase 2 infantry training than in combat and service support trades. Phase 3 training provides career progression courses for trained soldiers from the Field Army, both within their own trade, which may be required for promotion, and in new trades, and these soldiers may have operational experience.

In these types of training environments, within young populations that are exposed to the same marketing hype surrounding dietary and training supplements as civilian sportsmen and women, the use of supplements by some military personnel is to be expected. There is widespread use of supplements at all levels of civilian sport^(^ ^3^ ^)^ and a prevalence of 60–90 % supplement use is reported among high-performance UK athletes, including juniors (under 18 years of age)^(^ ^3^ ^–^ ^5^ ^)^. Supplements are readily available to the British military, both in and out of operational theatres, through a rapidly expanding market, and are regularly advertised in military publications.

Over the last 15 years, the importance of nutrition and feeding practices to military performance and operational readiness has gained increasing attention and support from within the British military, but the issue of self-administration of dietary and training supplements has been largely ignored. This has changed over the last 3 years or so, prompted by reports of adverse events related to supplement use in soldiers on operations and two subsequent studies by clinicians working in the theatre of operations in Iraq and Afghanistan^(^ ^6^ ^,^ ^7^ ^)^.

In the only published research that has sought to establish the point prevalence of dietary and training supplement use in British military populations, Boos *et al.* ^(^ ^6^ ^,^ ^7^ ^)^ studied supplement use among British soldiers on operations in Iraq during January 2009 and in a smaller cohort in Afghanistan during June 2010. In Iraq, the authors found a history of supplement use in 41 % of the respondents (*n* 417/1017), of which 32 % were current users and 9 % were past users. The most frequently given reasons for taking supplements were to increase muscle bulk (40 %) and to aid training and recovery (21 %). More recently in Afghanistan, 40 % of the respondents reported current use of supplements, but participant numbers in this study were small (*n* 87) and only personnel attending a health promotion fair were targeted. There is currently no reliable information related to the prevalence of dietary and training supplement use in UK-based British Army SuTs or soldiers.

Preliminary data from a cohort of Physical Training Instructor students (*n 378*), serving with various Corps within the Army, showed that 91 % of the respondents had been using some form of supplement within the past 12 months. Almost 50 % reported spending between £30 and 35 per month on supplements. Moreover, 51 % reported that they used more than one supplement at any given time and continued to use them while deployed on operations^(^ ^8^ ^)^. These findings indicate that supplement use among British military populations could be widespread and expensive and could have potential health, safety and operational implications. It is important to consider doping outcomes too, and the use of supplements may lead to a positive compulsory drug test with serious implications for a soldier's military career.

Therefore, the aim of the present study was to establish the point prevalence of supplement use in UK-based British Army SuTs and soldiers, to better inform the Chain of Command with regard to policy and educational needs.

## Materials and methods

### Participants

A total of 3054 male (96 %) and 114 female (4 %) British Army SuTs and soldiers who were based at UK training sites consented to take part in the present study. The present study was conducted according to the guidelines laid down in the Declaration of Helsinki, and an anonymous questionnaire was approved for use by the UK Ministry of Defence (MOD) Research Ethics Committee. Participants were recruited from eleven Phase 1, 2 and 3 training establishments, and the number of subjects (*n* 3168) equated to 3·1 % of regular Army strength.

Participant demographics and military ranks are given in Table 1. The respondents were serving with Infantry or Parachute Regiment Units (24 %), with the Royal Armoured Corps, Royal Artillery and the Royal Engineers (22 %), and with other Corps within the Army (51 %). The courses included basic military training for standard entrants and infantry (Phase 1), Phase 2 trade training, the Commissioning Course for Officer Cadets, and Phase 3 specialist promotional courses for Regular Army and Reserves, including physical training courses delivered by the Army School of Physical Training (ASPT).

**Table 1:** Demographics (*n* 3168) of all participants (all) and current supplement users (current users) (Number of subjects and percentages; mean values with standard deviations and ranges)

Characteristics	*n*	%
Sex (all)		
Male	3054	96
Female	114	4
Sex (current users)		
Male	1158	97
Female	40	3
Age (years) (all)		
Mean	22
sd	5
Range	16–52
Age (years) (current users)		
Mean	22
sd	5
Range	16–52
Rank (all, *n* 3166 responses)		
Phase 1 or 2 SuTs	1783	56
JNCO	564	18
OCdt	462	15
Pte	246	8
SNCO, WO and PS	111	3
Rank (current users)		
Phase 1 or 2 SuTs	592	49
JNCO	280	23
OCdt	137	11
Pte	127	11
SNCO, WO and PS	62	5
Physical activity levels (all, *n* 3155 responses)		
High	1899	60
Moderate	1212	39
Low	44	1
Physical activity levels (current users)		
High	844	71
Moderate	342	28
Low	8	1
Smoking status (all, *n* 2085 responses)		
Current	604	29
Ex	379	18
Never	1102	53
Smoking status (current users)		
Current	309	26
Ex	235	20
Never	646	54
Feeding (all, *n* 2078 responses)		
PAYD	955	46
DMR	813	39
Out of barracks	310	15
Feeding (current users)		
PAYD	574	48
DMR	433	37
Out of barracks	177	15
Supplement use		
Current	1198	38
Previous	1706	54

Phase 1 SuTs, Phase 1 soldiers under training; Phase 2 SuTs, Phase 2 soldiers under training; JNCO, Junior Non-Commissioned Officers, comprising Permanent Staff or personnel attending a Phase 3 Main Trade For Pay career progression course; OCdt, Officer Cadets undergoing officer training at the Royal Military Academy Sandhurst; Pte, private soldiers attending Phase 3 Main Trade For Pay career progression courses; SNCO, Senior Non-Commissioned Officers; WO, Warrant Officers; PS, Permanent Staff Officers; PAYD, Pay as You Dine, Service personnel pay at the point of service for the food they consume; DMR, Daily Messing Rate, the food provision is charged at a flat rate, which is deducted from a soldier's salary, and food is free of charge at the point of service.

Feeding arrangements varied between Pay as You Dine, Food Charge and Daily Messing Rate and other arrangements such as living and dining outside of barracks (Table 1). Under the Daily Messing Rate system, the food provision is charged at a flat rate, which is deducted from a soldier's salary, and food is free of charge at the point of service. Under the Pay as You Dine system, Service personnel pay at the point of service for the food they consume. Phase 1 training (which includes Officer Cadet training) comes under the Daily Messing Rate system.

A power calculation was performed using data from a recent study in which supplement use was surveyed in British soldiers on operations in Afghanistan during June 2010, using the point prevalence of supplement use as the end point^(^ ^7^ ^)^. A point prevalence of 40·2 % and an assumed non-response rate of 10 % indicated a minimum sample size of 2561, representing just over 2·5 % of current Army strength.

### Design

Data on the use of dietary and training supplements were collected using a purpose-designed, cross-sectional anonymous questionnaire, administered on a single occasion. Data collection took place from November 2010 to July 2011.

### Categorisation of dietary and training supplements

Dietary supplements are products that aim to supplement the diet and provide additional nutrients that may be missing from it or are not being consumed in sufficient amounts. For the purposes of the present study, vitamins and minerals, sports supplements, sports foods, stimulants and herbal products were considered to be dietary supplements. These products are often claimed to improve health and well-being and/or to sustain or improve some aspect of physical or mental performance.

In the present study, some supplements were grouped into exercise training supplements, and these include anabolic androgenic agents such as anabolic steroids and prohormones and peptide hormones and growth factors such as erythropoietin, insulins and growth hormone.

### Trial procedures

Permission to conduct the study was sought by Headquarters Army Recruiting and Training Division from the Commanding Officers of each training site under consideration. Once permission to proceed had been obtained, information sheets were distributed to potential participants, who were also fully briefed as a group by a trial investigator. During this briefing, details about the study, consent and the types of information to be requested were fully explained. It was made clear that participation in the study was voluntary, that individuals could not be identified, and that non-participation would not adversely affect their course outcome or military career. Each cohort was briefed by the same investigator, who wore civilian clothing and addressed potential participants as a civilian to emphasise the voluntary nature of the study. SuTs were targeted towards the end of their courses to better reflect supplement use during training. Potential subjects were given ample opportunity to ask questions during and after the briefing. No military staff allied to the SuTs was present during the data collection phase. Participation in the study approached 100 % of potential subjects. From a targeted population of 3171 personnel, 3168 (99·9 %) consented to take part in the study.

### Questionnaire

Data were collected using a purpose-designed, cross-sectional anonymous questionnaire. In Section 1 of the questionnaire, the participants were asked for demographic details including sex, rank range, training course and Arms/Service. Section 2 established normal physical activity levels, smoking status and daily feeding arrangements. The participants were asked whether and how often they currently used supplements, which supplements they currently used, how much was spent on supplements per month, how supplements were purchased and from where information on supplements was obtained. The participants were also asked whether they had used supplements in the past. A putative ban on supplement use existed within five of the eleven training establishments studied, but awareness of such bans was very mixed among personnel and within the command structure and supplements were often available from retail and vending outlets at these sites as well as from local amenities.

Current supplement users were asked to tick which supplements they used from the following list: (1) dietary supplements: creatine, amino acids, energy bars, Ca, fish oils, sodium bicarbonate, protein bars, powders and drinks, isotonic sports drinks, sports gels, folic acid, vitamin C, glucosamine, multivitamins, herbal remedies, e.g. ginseng, ginkgo, and echinacea, Fe tablets, antioxidants, and recovery drinks; (2) stimulants: caffeine tablets, caffeine gum, caffeine in tea, coffee and cola, caffeinated soft/sports drinks, cocaine, amphetamines and similar compounds, e.g. ephedrine; (3) exercise/training supplements: anabolic steroids, growth hormone, melatonin, erythropoietin, insulins and other anabolic agents, e.g. testosterone; (4) ibuprofen. The participants were also asked to indicate any other supplements used that were not listed.

Section 3 of the questionnaire aimed to establish the reasons for taking supplements and any concerns over the use of supplements. A total of seventeen questions were employed using a four-point forced scale (SA, strongly agree; A, agree; D, disagree; and SD, strongly disagree). Data were stored in accordance with the Data Protection Act 1998.

### Statistical analyses

Descriptive data were calculated as frequencies (%) and are presented as means with standard deviations for normally distributed variables. Logistic regression models were used to examine relationships between supplement use and the sex, age, course and smoking status of soldiers. OR were computed, and 95 % CI of the OR from these models are presented. Mann–Whitney *U* tests employing a Bonferroni-adjusted α were used to compare the prevalence of supplement use among personnel attending different courses. Pearson's χ^2^ test was used to compare types of supplements used according to course, rank, sex and smoking history of the participants. Statistical significance was accepted at *P <*0·05. All analyses were limited to current users of supplements, unless stated otherwise. Where percentages do not add up to 100, this is because data have been rounded to the nearest 1 %. Data in the text, tables and figures are presented as means with standard deviations. Data were analysed using SPSS version 18 (SPSS, Inc.).

## Results

### Participant characteristics

Details regarding the sex, age, smoking status, feeding arrangements and physical activity levels of the participants are given in Table 1. The respondents were split fairly evenly into those who considered that Army meals provide them with adequate energy (53 %) and those who did not consider so (47 %, NS), but a small majority considered that Army meals do not provide adequate nutrients (54 *v*. 46 %, *P*< 0·05).

### Supplement use

Overall, 38 % (*n* 1198; age 22 (sd 5) years) of the respondents reported current use of either dietary or training supplements or a combination of both and 54 % (*n* 1706; age 22 (sd 5) years) reported having taken supplements in the past 12 months. Of those who reported having taken supplements in the past 12 months, 21 % (*n* 352) had been deployed previously on operations.

The mean number of different dietary supplements taken by current users was 4·7 (sd 2·9), range 0–17, and the number of training supplements used was 0·1 (sd 0·3), range 0–1. Supplements were reportedly taken twice a day by 36 % of the users, on a daily basis by 34 %, every 2 d by 14 %, weekly by 9 %, and monthly by 2 % and 6 % indicated other. No current users selected ‘caffeine in tea, coffee and cola’ alone. Logistic regression modelling indicated that, for current users, younger age, being female, smoking and undergoing Officer Cadet training were associated with greater supplement use (Table 2).

**Table 2:** Association of the number of different supplements used with selected demographic characteristics of British Army soldiers under training and soldiers based at UK Phase 1, 2 and 3 training establishments (*n* 1198 current users)† (Odds ratios and 95 % confidence intervals)

Characteristics	Six to ten supplements	Eleven to twenty supplements
	OR	95 % CI	OR	95 % CI
Sex				
Male	1·0		1·0	
Female	0·93	0·42, 2·06	3·39*	1·33, 8·70
Age (years)				
16–20	0·32**	0·16, 0·66	0·09***	0·02, 0·33
21–25	0·52	0·27, 1·01	0·26*	0·09, 0·80
26–30	0·60	0·30, 1·19	0·32	0·10, 1·01
>30	1·0		1·0	
Smoking status				
Current	1·25	0·90, 1·74	2·13*	1·04, 4·36
Ex	1·02	0·71, 1·47	1·71	0·83, 3·52
Never	1·0		1·0	
Course				
SuTs Phase 1	1·01	0·46, 2·22	2·86	0·50, 16·45
SuTs Phase 2	1·08	0·51, 2·29	1·53	0·26, 8·99
Phase 3	0·88	0·40, 1·94	1·92	0·33, 11·23
Ocdt (RMAS)	1·84	0·83, 4·11	7·23*	1·28, 40·72
ASPT	1·44	0·69, 2·98	2·21	0·42, 11·77
P Coy	1·22	0·46, 3·26	1·48	0·12, 18·74
PS	1·0		1·0	

SuTs Phase 1, Phase 1 soldiers under training; SuTs Phase 2, Phase 2 soldiers under training; Phase 3, Main Trade For Pay career progression course; OCdt, Officer Cadets undergoing officer training at the Royal Military Academy Sandhurst; RMAS, Royal Military Academy Sandhurst; ASPT, Army School of Physical Training; P Coy, Pegasus Company Pre-Parachute Selection; PS, Permanent Staff.

Values were significantly different between the reference level and other levels within a given characteristic: * *P*< 0·05; ** *P*< 0·01; *** *P*< 0·001.

† Reference category is ‘currently taking one to five supplements’.

Among the respondents, 248 (equating to 8 % of the respondents or 21 % of the current users) spent less than £10 per month on supplements, 219 (7, 18 %) spent £10–20, 189 (6, 16 %) spent £20–25, 249 (8, 21 %) spent £25–35, and 286 (9, 24 %) spent more than £35. Altogether, 45 % of the current users spent more than £25 per month. The range of supplements used increased with increased spend (*P*< 0·05).

### Types of supplements used

**Fig. 1:**
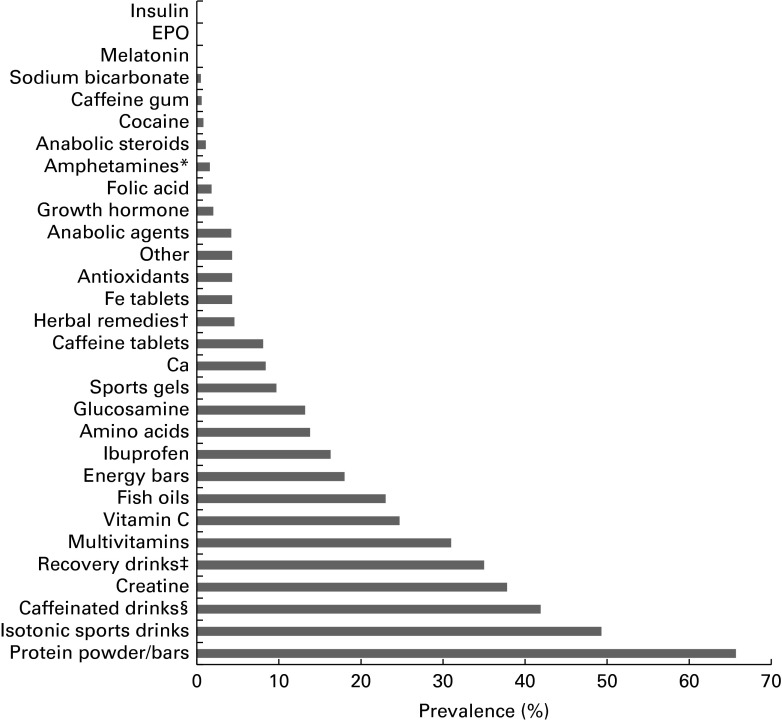
Supplements used by British Army soldiers under training and soldiers based at UK Phase 1, 2 and 3 training establishments (*n* 1198 current users). * Amphetamines and other compounds including ephedra and ephedrine alkaloids. † Herbal preparations such as ginseng, ginkgo and echinacea. ‡ Recovery drinks including Lucozade Sport Recovery and Recovermax. § Caffeinated drinks including Relentless, Red Bull and Lucozade Sport Caffeine Boost, excluding caffeine in tea, coffee and cola.

The most commonly used dietary supplements among current users were protein bars, powders and drinks, including whey protein (66 %), isotonic carbohydrate–electrolyte sports drinks (49 %), creatine (38 %), recovery sports drinks (35 %), multivitamins (31 %) and vitamin C (25 %) (Fig. 1).

Caffeine was widely consumed in the form of caffeinated drinks (42 %), such as Relentless, Red Bull, Monster and Lucozade Sport Caffeine Boost, and in tea, coffee and cola (47 %). Caffeine use in tea, coffee and cola and the use of caffeinated drinks were found to be higher in current smokers (53 and 54 %, respectively) than in those who had never smoked (45 %, *P*< 0·05; 36 %, *P*< 0·001, respectively).

A small number of current users reported the use of amphetamines and similar compounds (*n* 19, 1·6 %), cocaine (*n* 9, 0·8 %), anabolic steroids (*n* 13, 1·1 %), growth hormone (*n* 24, 2·0 %) and other anabolic agents, e.g. testosterone (*n* 50, 4·2 %). The use of ibuprofen was reported by an average of 16 % of the respondents. There was little reported use of folic acid (1·8 %), Ca supplements (8·4 %), sodium bicarbonate (0·5 %), Fe tablets (4·3 %) or caffeine tablets (8·1 %) across the ranks. The use of Ca and Fe tablets was higher in females (28 and 18 %, respectively) than in males (8 %, *P*< 0·001; 4 %, *P*< 0·001, respectively).

### Supplement use by rank and course

There was no difference in the prevalence of supplement use between personnel in junior ranks (SuTs, private soldiers attending Phase 3 Main Trade For Pay career progression courses and Junior Non-Commissioned Officers (JNCO); 39 %) and those in senior ranks (Senior Non-Commissioned Officers (SNCO), Warrant Officers, Commissioned Officers and Officer Cadets; 35 %; NS).

There was a significant difference in the prevalence of supplement use between personnel attending courses and Permanent Staff (PS, *P*< 0·001). The prevalence of supplement use varied according to the course attended, with less than one-third of soldiers undergoing Phase 1 training and almost three-quarters of those attending the Pegasus Company Pre-Parachute Selection (P Coy) course reporting supplement use (28, 30, 39, 42, 55, 56 and 74 % of those attending Phase 1, Officer Cadet (RMAS), Phase 2, Phase 3, PS, ASPT (eight different courses) and the P Coy courses, respectively). There was a significant difference in the prevalence of supplement use between soldiers undergoing Phase 1 training and those attending all courses except Officer Cadet training at the RMAS; between soldiers undergoing Phase 2 training and those attending all courses except Phase 3 courses and Officer Cadet training at the RMAS; between soldiers attending Phase 3 courses and those attending all courses except Phase 2 training and PS; between soldiers undergoing Officer Cadet training at the RMAS and those attending all courses except Phase 1 and 2 training; between soldiers attending the P Coy course and those attending all courses except ASPT and PS; between PS and those attending all courses except Phase 3 and P Coy courses; and between those attending ASPT courses and those attending all courses except P Coy and PS (all *P*< 0·05).

There were significant differences in the types of supplements used among personnel in different ranks. JNCO reported a greater use of amino acid products (23 % of current users; *P*< 0·001) and anabolic agents (8·9 %; *P*< 0·001) compared with personnel in any other rank. They also reported greater use of dietary protein products compared with personnel in other ranks (75 %, *P*< 0·001). Creatine use was significantly higher in SuTs than in JNCO (40–44 %, *P <*0·001), with the exception of Officer Cadets, whose usage was lower than that of personnel in all other ranks (18 %, *P*< 0·001). SuTs and JNCO were more likely than personnel in other ranks to use caffeinated drinks (49 and 40 %, respectively, *P <*0·001).

SNCO, Officers and Officer Cadets were more likely than personnel in other ranks to use energy bars (28, 46 and 33 % of the current users, respectively; *P <*0·001), vitamin C (30, 32 and 38 %, respectively; *P <*0·05) and sports gels (35, 32 and 22 %, respectively; *P <*0·001). SNCO were more likely than personnel in other ranks to use fish oils (45 %, *P <*0·001), and Officer Cadets were more likely than personnel in other ranks to use isotonic sports drinks (72 %, *P*< 0·001), multivitamins (52 %, *P*< 0·001), vitamin C (*P*< 0·05), glucosamine (33 %, *P*< 0·001), herbal remedies (11 %, *P*< 0·05), caffeine in tea, coffee and cola (73 %, *P <*0·001) and ibuprofen (39 %, *P <*0·001).

Soldiers attending a Phase 3 Main Trade For Pay course reported the greatest use of creatine (48 % of the current users) compared with those attending all other courses (range 0–53 %, *P <*0·001). Those attending courses at the ASPT reported the greatest use of amino acids and protein products (*P <*0·001). The use of caffeinated drinks was highest in soldiers undergoing Phase 1 (48 %) and Phase 2 (50 %) courses, compared with those attending other courses. The prevalence of ibuprofen use was 2-fold to 3-fold higher in personnel undergoing the P Coy course (Infantry; 41 %, *P <*001) and those undergoing Officer Cadet training (40 %, *P <*001) compared with those attending other courses (range 11–14 %).

### Supplement use by sex

Analysis of sex differences in supplement use is hampered by the small number of females in the study (*n* 114; current users *n* 40), and the results should be interpreted with caution. In the present study, it was found that males were more likely than females to use creatine (39 and 5 %, respectively; *P <*0·001) and protein bars, powders and drinks including whey protein (67 and 20 %, respectively; *P <*0·001). By comparison, females were more likely than males to use multivitamins (65 and 30 %, respectively; *P <*0·001), glucosamine (40 and 12 %, respectively; *P <*0·001), fish oils (48 and 22 %, respectively; *P <*0·001), caffeine in tea, coffee and cola (75 and 46 %, respectively; *P <*0·001), and ibuprofen (45 and 15 %, respectively; *P <*0·001). Logistic regression analysis showed a significant relationship between being female and higher supplement use (*P <*0·05; Table 2). There were no sex differences in the use of energy bars, isotonic sports drinks and recovery drinks, and there were too few cases for analysis of the use of Ca, sodium bicarbonate, sports gels, folic acid, herbal remedies, caffeine tablets, antioxidants and training supplements.

### Reasons for use

The questionnaire sought to establish the reasons for taking supplements using a four-point forced scale (SA, strongly agree; A, agree; D, disagree; and SD, strongly disagree; Table 3). The main reasons given for taking supplements were as follows: to recover from training or physical activity (91 % agree or strongly agree); to improve physical performance (87 %); to prepare for a period of training or physical activity (86 %); to supplement the diet (74 %; Table 3). Most of the respondents agreed that they had a very good understanding of why they took supplements and did not believe that they had suffered side effects from taking supplements. Only about one-third of the respondents reported taking supplements for health reasons or to reduce their risk of becoming ill. Even fewer reported taking supplements for medical reasons or to help lose weight.

**Table 3:** Reported reasons for use of supplements, and experience of side effects, in British Army soldiers under training and soldiers based at UK Phase 1, 2 and 3 training establishments (Number of responses and percentages)

Reasons for supplement use	*n*	Strongly agree (%)	Agree (%)	Disagree (%)	Strongly disagree (%)	Totally agree (strongly agree +agree) (%)	Totally disagree (strongly disagree+disagree) (%)
I take supplements to top up my diet	1190	30	44	20	6	74	26
I take supplements to top up ORP in the field	1111	10	26	44	21	36	64
I take supplements to improve physical performance	1189	45	42	10	3	87	13
I take supplements to help me prepare for a period of training or physical activity	1189	40	46	11	3	86	14
I take supplements to help me recover from training or physical activity	1189	49	42	7	2	91	9
I take supplements to improve my mental performance and/or to help me stay awake	1187	15	30	39	16	46	54
I take supplements for health reasons	1183	11	25	43	22	35	65
I take supplements for medical reasons	1184	3	9	53	36	11	89
I take supplements to help lose weight	1181	5	13	42	40	18	82
I take supplements to gain weight	1184	14	32	31	23	47	53
I take supplements to reduce my risk of injury	1182	11	33	39	18	44	57
I take supplements to reduce my risk of becoming ill	1187	9	25	43	23	34	66
I take supplements because I am told that supplements can help me	1186	13	41	31	16	53	47
I have a very good understanding of why I take, or have taken, supplements	1186	39	52	7	2	91	9
I believe that I have suffered side effects from taking supplements	1184	3	10	40	47	13	87
I have no concerns about the potential side effects of using supplements	1193	21	39	29	11	60	40

ORP, UK operational ration packs.

### Information and purchasing

**Fig. 2:**
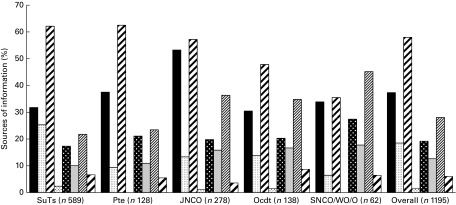
Sources of information about supplements used by British Army SuTs and soldiers based at UK Phase 1, 2 and 3 training establishments. SuTs, Phase 1 or Phase 2 soldiers under training; Pte, private soldiers attending Phase 3 Main Trade For Pay career progression courses; JNCO, Junior Non-Commissioned Officers, comprising Permanent Staff or personnel attending a Phase 3 Main Trade For Pay course; OCdt, Officer Cadets undergoing officer training at the Royal Military Academy Sandhurst; SNCO, Senior Non-Commissioned Officers; WO, Warrant Officers; O, Permanent Staff Officers. 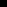, Internet; 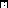, TV; 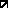, friend; 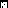, radio; 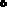, health care professional; 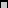, print media; 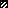, sports science articles; 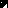, other.

The majority of all respondents (82 % agree or strongly agree) and current users (85 % agree or strongly agree) agreed that more education should be provided regarding supplements. The most popular sources of information were friends and the Internet, followed by sports science articles, television, health care professionals (including Physical Training Instructor (PTI)), print media including newspapers, radio and other sources (Fig. 2).

**Fig. 3:**
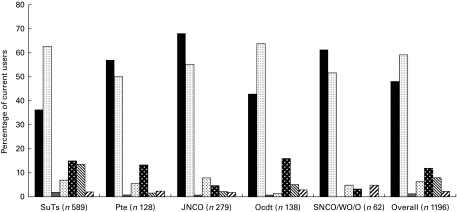
Routes of purchase for supplements used by British Army SuTs and soldiers based at UK Phase 1, 2 and 3 training establishments. SuTs, Phase 1 or Phase 2 soldiers under training; Pte, private soldiers attending Phase 3 Main Trade For Pay career progression courses; JNCO, Junior Non-Commissioned Officers, comprising Permanent Staff or personnel attending a Phase 3 Main Trade For Pay course; OCdt, Officer Cadets undergoing officer training at the Royal Military Academy Sandhurst; SNCO, Senior Non-Commissioned Officers; WO, Warrant Officers; O, Permanent Staff Officers. 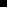, Internet; 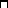, local amenities; 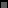, print media; 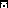, given by friend; 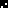, onsite retail outlets; 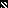, vending; 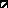, other.

The most popular points of purchase for supplements were local shopping amenities and the Internet, followed by onsite retail outlets (Navy, Army and Air Force Institutes; NAAFI), onsite vending machines, friends and print media (magazines, etc.; Fig. 3).

## Discussion

This is the first study to investigate the prevalence of dietary and training supplement use in UK-based British military personnel. The majority of subjects in the present study were British Army SuTs, 91 % of whom were aged ≤ 25 years, engaged in arduous military training, the results of which determine their career progression through the Field Army. The major finding of the present study was a prevalence of 38 % supplement use within this population. This is consistent with a prevalence in the range of 30–40 % reported in operational British Army personnel by Boos *et al.* ^(^ ^6^ ^,^ ^7^ ^)^, but these earlier findings do not reflect the varying prevalence of use between Army trades.

In the present study, a variation was observed in the prevalence of supplement use, with one-third of Phase 1 SuTs, more than half of ASPT students and almost three-quarters of the Parachute Regiment undertaking the P Coy course, the arduous selection tests in weeks 19 to 20 of training, reporting supplement use. The training undergone during P Coy course is physically and mentally demanding and is designed to ‘push candidates to their limits and beyond’^(^ ^9^ ^)^, indicating that, as expected, the prevalence of supplement use may be associated with the physical demands placed on individuals.

Variation in supplement use between Army trades is reflected in the military literature. In a defence-wide survey of active-duty US military personnel, for instance, 60 % reported the use of supplements^(^ ^10^ ^,^ ^11^ ^)^, and a similar percentage was reported for the wider US Army, in which 53 % of soldiers surveyed reported using supplements at least once a week^(^ ^12^ ^)^; this increased to more than 80 % of US Army Rangers^(^ ^13^ ^,^ ^14^ ^)^ and 87 % of US Army Special Forces^(^ ^15^ ^)^, which, in support of the present study, is likely to reflect the arduous nature of their occupation.

Another finding of the present study was that a wide range of supplements were self-administered by British military personnel in training and that these included not just common nutritional supplements such as protein powders, sports drinks, creatine and vitamins, and common stimulants such as caffeine, but also anabolic androgenic steroids, growth hormone and stimulants such as cocaine and amphetamines or similar compounds.

The most commonly used dietary supplements were protein products, isotonic carbohydrate–electrolyte and recovery sports drinks, caffeinated drinks, creatine and multivitamins. Altogether, 45 % of the current users spent more than £25 per month on supplements, indicating that these are purchased at considerable expense to users surveyed in the present study.

In the present study, the reported use of amphetamines and/or similar compounds (one in 167 personnel), cocaine (one in 352), anabolic androgenic steroids (one in 244), growth hormone (one in 132) and other anabolic agents (one in 63), e.g. testosterone, was low, but this indicates that the range of stimulants and training supplements in use during periods of training in the UK is at least as great as that reported by soldiers on deployment^(^ ^6^ ^,^ ^7^ ^)^. The incidence of anabolic steroid use found in the present study exceeds that in Afghanistan^(^ ^7^ ^)^, but it is almost 3-fold lower than that reported by soldiers on deployment in Iraq a year earlier^(^ ^6^ ^)^. The present study did not establish the entry point for the use of training supplements by military personnel.

The use of anabolic agents and prohormones is prohibited under Service law and under the terms of the World Anti-Doping Code that is endorsed by the UK Government through its National Anti-Doping Organisation (UK Anti-Doping). The use of anabolic agents is monitored as part of the Armed Forces Compulsory Drugs Testing process, and a positive compulsory drug test may result in discharge. The supply of anabolic agents is a criminal offence under UK law. The issue of how far to extend the prohibition of supplement use within the military is an interesting one. Unlike in sport, the military does not seek to create a level playing field and should seek advantages where it can, subject to appropriate risk–benefit analyses. Research and information on human performance have been available to military health professionals for many years through the Technical Co-Operation Programme, an international Defence organisation, but have never been widely disseminated or used in the UK.

There is neither a sound rationale nor supporting evidence for many of the supplements available to personnel. There are, however, probably two scenarios in which some supplements might confer an advantage. First, in accordance with International Olympic Committee advice to athletes^(^ ^16^ ^)^, although supplements should not be used to compensate for poor food choices and an inadequate diet where a choice exists, supplements that provide additional energy and/or essential nutrients may be useful when food intake or food choices are restricted for extended periods for reasons including operational constraints, extended travel, and periods when preparation and/or consumption of adequate meals or operational rations is not possible or desirable. Fortification of UK operational rations with vitamins, albeit limited, ceased in 2010 when the MOD food contract was awarded to an external contractor. This was done on the basis that the statement of requirement for operational rations had been revised to meet UK Military Dietary Reference Values^(^ ^17^ ^)^, without the need for further fortification^(^ ^18^ ^)^.

In support of a role for dietary supplements, it has been demonstrated that a daily mixed nutritional supplement, sufficient to offset two-thirds of the estimated energy deficit during an 8-week arduous Phase 3 military training programme, can attenuate decreases in body mass and lean mass, protect immune function and prevent the decrease in physical performance observed without supplementation^(^ ^19^ ^,^ ^20^ ^)^. Moreover, studies of US Ranger training have shown that a small carbohydrate-based supplement equivalent to 1·7 MJ/d, which reduced mean energy deficit from 31 to 24 % over the 8-week training period, while unable to arrest the decline in dynamic muscle strength, was associated with a decrease in the percentage of soldiers treated for infections from 25 and 24 to 8 and 2 % during the mountain and jungle phases of training, respectively^(^ ^21^ ^)^. Moreover, increasing the carbohydrate content of the diet to a tolerable level (approximately 8·5 g carbohydrate/kg per d (approximately 65 % of total energy intake)) during a period of intensified running training has been shown to better maintain physical performance and mood state, thereby reducing the symptoms of overreaching^(^ ^22^ ^)^.

Second, a small number of supplements have proven benefits to physical and/or cognitive performance when used in accordance with current evidence. These include creatine, caffeine, carbohydrate supplements, carbohydrate/protein recovery supplements, and muscle-buffering agents such as sodium bicarbonate, sodium citrate and β-alanine, and possibly l-arginine and nitrates (for reviews, see Maughan *et al.* ^(^ ^3^ ^)^, Burke *et al.* ^(^ ^23^ ^)^). As well as the Technical Co-Operation Programme output, guidelines for the use of bespoke caffeine products by military personnel to enhance physical and cognitive performance and combat readiness have been available since 2004^(^ ^24^ ^)^.

The role of supplements in attenuating the rate and extent of fatigue and the degradation of physical and cognitive performance that occurs as a consequence of arduous training and/or exposure to environmental stressors, including heat, cold and high altitude, as well as psychological stress and sleep deprivation, should be considered carefully by the UK military. It is the opinion of the authors that the UK military should continue to take a more considered stance on the use of supplements in the same manner as UK Sport^(^ ^25^ ^)^ and the International Olympic Committee^(^ ^16^ ^)^, as reflected in the recent tri-service policy statement on supplement use issued by the Surgeon General's Department^(^ ^26^ ^)^. Military personnel who compete in elite sport and are subject to drug testing should continue to be directed to the World Anti-Doping Code 2011 Prohibited List for substances and methods prohibited at all times in and out of competition.

The most popular reasons given for taking supplements in the present study were as follows: to recover from training or physical activity; to improve physical performance; to prepare for a period of training or physical activity; to supplement the diet. Few subjects reported having experienced side effects from supplement use or using supplements for medical reasons or to help lose weight. Approximately two-thirds reported that they did not take supplements for health reasons or to reduce the risk of becoming ill, and most of them reported having a very good idea about why they took supplements. These findings are consistent with the findings of other studies of UK military personnel^(^ ^6^ ^)^, but differ somewhat from those of US Army surveys of both male and female soldiers who rated health as the primary reason for taking supplements^(^ ^14^ ^)^. Likewise, a study by the UK Food Standards Agency, which found that one-third of people in the UK take some dietary supplement, the majority on most days, suggests that health and well-being are the primary motives for civilian use of supplements^(^ ^27^ ^)^. This confirms observations made over the years by the lead author, suggesting that, broadly speaking, the populations investigated in the present study focus to a far greater extent on the effects of nutrition and supplements on training and recovery than on longer-term health outcomes.

Reasons for supplement use given in the present study are broadly similar to the profiles of young elite athletes in the general population in terms of an emphasis on training and recovery^(^ ^28^ ^)^, but differ in a greater focus on supplementing the daily diet and maintaining cognitive function and wakefulness. The most likely explanations for this are the more regimented in-barrack feeding and sleeping arrangements of the majority of participants in the present study, who have fewer opportunities to meet individual dietary and sleep needs and preferences than young civilian athletes, and the intensive nature of the courses that combine physical training with academic instruction.

Guidance on supplement use was found to be inconsistent across the Army training establishments. A putative ban on supplement use had been imposed by some training sites, mainly Phase 1 and 2 sites, including the RMAS, but not by others, and awareness and observation of such bans were very mixed among personnel and within the command structure. Moreover, supplements were often advertised and sold from retail and vending outlets at these sites as well as from local amenities. What generally appears to be the default position of training sites to ban the use of all dietary supplements and performance-enhancing substances is understandable and advisable (1) in the absence of independent, reliable, evidence-based information related to supplements, (2) in relation to illegal or harmful substances and those prohibited under Service law, and (3) where there exists a concern over contamination. In reality, it is likely that such bans have been introduced in the past because this is an area of training over which it is difficult to exert control or issue advice in the absence of reliable information or training for decision makers, including medical staff.

The present study demonstrated that the most popular source of guidance on supplement use for current users among Phase 1 and Phase 2 SuTs was their friends, rather than a health care professional or PTI, with whom they generally spend time in a formal setting. The Internet generally is also less accessible to SuTs compared with other groups due to constraints of time and resources, which may explain the lower reported use of online information compared with personnel in other ranks. In an unpublished study of feeding practices of Officer Cadets at RMAS in 2009, however, we found that PTI are a potentially important and accessible point of contact for Cadets seeking nutrition- and supplement-related advice, which is given informally. PTI reported that they are frequently asked for advice regarding supplements to optimise performance, including protein products and creatine, and supplements to be taken as part of their normal daily diet. The advice given by PTI is, by their own admission, based largely on individual opinions, experiences and personal training. Advice is not consistent or the result of specialised education or training, routinely accessing independent scientific advice, or a reflection of RMAS or Army policy in this area^(^ ^29^ ^)^.

In accordance with current International Olympic Committee (IOC) advice^(^ ^16^ ^)^ and recently established military policy^(^ ^26^ ^)^, military personnel contemplating the use of supplements should consider their efficacy, their cost, the risks to health or performance, and the potential for a positive compulsory drug test. It is the view of the authors that self-administration of dietary and training supplements by juniors and by female Army personnel, for example, in relation to immune function and bone and joint health, as well as the potential for a positive role for proven supplements in training and combat environments, should be examined further. The entry point for the use of prohibited training supplements by military personnel in the UK and those on operations, as well as the prevalence of supplement use among soldiers on operations and among those returning to the UK from current operations, also requires further examination.
